# Identification of unique B virus (*Macacine Herpesvirus 1*) epitopes of zoonotic and macaque isolates using monoclonal antibodies

**DOI:** 10.1371/journal.pone.0182355

**Published:** 2017-08-04

**Authors:** David Katz, Wei Shi, Manjunath S. Gowda, Mugdha Vasireddi, Irina Patrusheva, Hyuk-Kyu Seoh, Chadi N. Filfili, Martin J. Wildes, Jay Oh, Julia K. Hilliard

**Affiliations:** 1 Department of Biology, Viral Immunology Center, Georgia State University, Atlanta Georgia, United States of America; 2 Department of Biology, GSU Biology Proteomics Core Facility, Georgia State University, Atlanta Georgia, United States of America; University of California Irvine Medical Center, UNITED STATES

## Abstract

Our overall aim is to develop epitope-based assays for accurate differential diagnosis of B virus zoonotic infections in humans. Antibodies to cross-reacting epitopes on human-simplexviruses continue to confound the interpretation of current assays where abundant antibodies exist from previous infections with HSV types 1 and 2. To find B virus-specific epitopes we cloned ten monoclonal antibodies (mAbs) from the hybridomas we produced. Our unique collection of rare human sera from symptomatic and asymptomatic patients infected with B virus was key to the evaluation and identification of the mAbs as reagents in competition ELISAs (mAb-CE). The analysis of the ten mAbs revealed that the target proteins for six mAbs was glycoprotein B of which two are reactive to simian simplexviruses and not to human simplexviruses. Two mAbs reacted specifically with B virus glycoprotein D, and two other mAbs were specific to VP13/14 and gE-gI complex respectively. The mAbs specific to VP13/14 and gE-gI are strain specific reacting with B virus isolates from rhesus and Japanese macaques and not with isolates from cynomolgus and pigtail macaques. The mAb-CE revealed that a high proportion of naturally B virus infected rhesus macaques and two symptomatic humans possess antibodies to epitopes of VP13/14 protein and on the gE-gI complex. The majority of sera from B virus infected macaques and simplexvirus-infected humans competed with the less specific mAbs. These experiments produced a novel panel of mAbs that enabled B virus strain identification and confirmation of B virus infected macaques by the mAb-CE. For human sera the mAb-CE could be used only for selected cases due to the selective B virus strain-specificity of the mAbs against VP13/14 and gE/gI.

To fully accomplish our aim to provide reagents for unequivocal differential diagnosis of zoonotic B virus infections, additional mAbs with a broader range of specificities is critical.

## Introduction

B virus (*Macacine herpesvirus 1*) is a member of the family *Herpesviridae* in the genus *Simplexvirus* within the subfamily *Alphaherpesviridae* [1–5]. Primary B virus infections in the natural host (macaques), establish a latent infection in the sensory dorsal root or cranial ganglia subserving the regions of the original inoculation site(s). Stress-induced reactivation is accompanied at times unpredictable events of virus-shedding detectable from mucosal surfaces. When there are symptoms, these are often mild and transient unless the immune sytem is compromised. Cross-species B virus infections are associated with enhanced virulence resulting in serious clinical disease and frequent mortality in nonhuman primates as well as in zoonotic infections [2–9]. Fatality rate in untreated humans may reach 80% in the absence of timely interventions. Humans surviving infection can harbor B virus latently and can suffer reactivation. Symptomatic reactivation of B virus has been documented in latently infected humans [10, 11], however, there are at least several more cases, which have not been published but these were documented clinically and with laboratory evaluations (Hilliard, unpublished communication)

Early accurate diagnosis of B virus infections in macaques, non-human primates, and humans is critical to contain infection, and in cases of human zoonotic infection enables early antiviral intervention to prevent fatalities. Because virus shedding is unpredictable, reliance on direct virus detection techniques is impractical, thus diagnosis is based mainly on serology [12, 13].

For diagnosing B virus infection in macaques in the National B Virus Resource Laboratory we use a titration ELISA (tELISA) and one or more of three confirmatory tests: western blot analysis (WBA), the recombinant-based ELISA (Rec-ELISA), and competition ELISA (cELISA) [12–15]. B virus antigens used in these assays cross react with other simplexviruses. These tests are sufficient for diagnosing B virus infections in macaques, because no other cross-reacting viruses are known to infect them [1, 5, 12]. However, in humans B virus diagnosis is confounded by potential co-infection with two cross-reacting human simplexviruses, HSV-1 and/or HSV-2. To overcome this problem, B virus specific antigens (epitopes) that are currently not available are needed.

Optimum tools for specific epitope identification included monoclonal antibodies (mAbs) that can be used as reagents in competition ELISAs [16, 17] or in combination with technologies using phage-display peptide libraries or overlapping peptide-arrays [18–20].

Monoclonal antibodies to B virus antigens were produced in the past by other investigators. Some of the mAbs were highly B virus specific but their use was mostly limited to the identification of BV isolates and for macaque serology [16, 17, 21, 22].

Several strategies can be used for the production of specific mAbs including using synthetic peptides with predetermined specificity. However, one of the most powerful features of the monoclonal antibody production method that enables the study of *complex antigenic structures* is that specific monoclonal antibodies can be recovered using non-purified immunogens. This approach is independent of software predictions (that are not always accurate) and enables selection of antibodies not only to linear epitopes but also to conformational epitopes.

In this study we have chosen to use this strategy to produce mAbs to whole B virus native proteins allowing the immune system of the mouse to select the optimal epitopes that will enable differential diagnosis of B virus infections in humans.

Here, we describe the characterization of a novel panel of mAbs that were produced by inoculating mice with B virus and recombinant glycoprotein B (gB) immunogens.

## Materials and methods

### Virus, Antigens and cells

Stocks of five B virus isolates were prepared by infecting African Green Monkey kidney cells (Vero) (ATCC® CCL-81™, Manassas, VA), as previously described for the B virus laboratory strain E2490 [12]. Isolates include E2490 laboratory strain (RH-BV) derived from rhesus macaque (*Macaca mulata*), a gift from the late Dr RN Hull, Eli Lilly, Indianapolis IN, clinical isolates, RRn6, isolated from a rhesus macaque (JH lab), Pmn1 (PT-BV) isolated from a pigtail macaque (*Macaca nemistrina*) (JH lab), CQ8166 (Cyno-BV) isolated from a cynomolgus macaque (*Macaca fascicularis*) (JH Lab), and 10R (Jap-BV) isolated from a Japanese macaque (*Macaca fuscata*) (JH lab). Propagation and harvesting of B virus isolates were performed in the GSU BSL4 Laboratory in accordance with the guidelines of the 5^th^ edition of Biosafety in Microbiological and Biomedical Laboratories [12]. Stocks of other simplexviruses, HVP2 (*Papiine herpesvirus* 1) (EOUP-16) from baboons (*Papio spp*), HVL (isolated by JKH lab) from a langur monkey (*Presbytis spp*), HVM (isolated from a sooty mangabeys (*Cercocebus atys*) (JKH lab), SA8 (*Cercopithecine herpesvirus* 2) (B264) isolated from African Green Monkeys (*Cercopithecus aethiops*), HSV-1 (KOS), and HSV-2 (186), were prepared similarly in Vero cells (ATCC CCL-81) under standard BSL2 conditions as previously described [1, 12]. Virus titers determined by a standard plaque assay ranged between 10^9^ and 10^10^ plaque forming units per milliliter (PFU/ml). Virus-antigens for ELISA were prepared by solubilization in mixture of Tween-40 and sodium deoxycholate (Sigma Chemical, St Louis, Mo) as previously described [12, 23], except that the picric acid step was omitted. Control antigens for ELISA were prepared from detergent lysates of passage-matched uninfected Vero cells (UN antigen) as described for virus-antigens [1, 12]. Antigens for WBA were prepared by adding 1% SDS to pellets of B virus-infected cells immediately after harvesting [12]. The protein concentrations of antigen preparations were evaluated by the Thermo Scientific Pierce BCA Protein Assay kit (Rockford, IL). The reactivity of antigens was assessed in comparison with previous antigen and antibody lots and stored at –70°C or lower. B virus recombinant gB, gC, gD, gE, gG and gI as well as HSV-1 recombinant gB and gD were prepared as previously described [15].

### Serum sample collection

Nonhuman primate sera and human sera were obtained from the National B Virus Resource Center (Atlanta, GA). Diagnoses of a B virus, HSV -1, and HSV-2 infection were based on all data available including clinical history and laboratory tests (tELISA, WBA, competition ELISA, recombinant ELISA, cell-culture isolation and/or PCR) [12, 13].

### Preparation of B virus immunogen in mouse cell-cultures

To limit the amount of antigens that are foreign to the mouse, we propagated rhesus B virus E2490 stock (RH-BV) in mouse 3T3 fibroblast cells (NIH/3T3 (ATCC® CRL-1658™).

The virus was grown in two 850 cm^2^ roller bottles in DMEM high glucose supplemented with 10% FBS, 200 mM L-glutamine and antibiotics (Penicillin/Streptomycin) at 37°C. Confluent cell monolayers (95%) in the roller bottles were infected with either one of the B virus strains (5 MOI) and maintained in DMEM high glucose supplemented with 1% FBS and antibiotics. The infected cells were incubated for 24 hrs at 34°C, scraped into the media and centrifuged at 800 Xg for 10 min. Cell pellets were resuspended in 4.5 ml of sterile ultrapure water. The suspension was then sonicated using Sonics Vibra Cells Sonicator at 75% Amp for 5 min. Cell debris was removed by centrifugation (800 Xg/10 min) and the virus suspension (about 5 ml) was saved. The final virus titer was determined by the standard plaque assay as 2x10^7^ PFU/ml. For inactivation of the B virus preparation we have used a “Psoralen- Broad Spectrum Light Pulses (BSPL) Photoinactivation Technique.”

### The psoralen-BSPL photoinactivation technique (Psoralen-BSPL)

Unlike detergent inactivation the psoralen photoinactivation damages virus nucleic acids, thus potential viral DNA replication in the inoculated mice is eliminated [23–25].

The Psoralen- Broad Spectrum Pulsed Light (BSPL) technique for B virus inactivation was performed as described in the “U.S. Provisional Patent Application. Title: Photo-Inactivated Viruses And Systems and Methods of Using The Same Serial No.: 61/288,756. Filing Date: 21 December 2009. GSURF Ref. No.: 2009–01. TS Ref. No.: GSU200901PRV”. Publication no. WO/2011/084748, April 14, 2011. Seventy μl of 2 mg/ml psoralen (4-aminomethyl-trioxsalen hydrochloride, # A4330, Sigma) were added to 7 mL of a 1:5 dilution of the virus suspension (4x10^6^ PFU/ mL) resulting in psoralen final concentration of 20 μg/ml. The virus-psoralen mixture was transferred in 1 ml portions to polyethylene tubings (Polyethylene (Low Density) polytubing # S-3520, 1" wide, 2 Mil Poly Tubing Roll, ULINE, Atlanta GA) that were heat sealed at one end. After transferring the virus to the tubing the other end was heat sealed at a distance of 5 cm from the opening. After sealing, the tubing was inserted into a 3MIL thick polyethylene bag and sealed at both ends. Briefly, for BSPL exposure, these sealed polyethylene tubings, were placed on a flat bed of ice on a tray and inserted into the irradiation chamber of the SteriPulse-XL device (RS-3000C, Xenon Corp.) and exposed to 12 pulses/4 seconds of BSPL that sums up to a total energy of 5.4 jouls/cm2. Following irradiation the contents of each individual tubing were pooled and tested for the presence of residual B virus by an infectivity test in Vero cells, for DNA damage by PCR, and for antigenicity by ELISA.

#### Infectivity test in Vero cells for validating the inactivation of B virus

Pooled B virus suspension (500 μL) was tested for infectivity in Vero cell monolayers grown in 6-well plates. The cultures were observed microscopically for B virus cytopathic effect (CPE) for 48 hrs. If no CPE developed, cells from the virus infected well were then scraped and transferred to another well containing Vero cells for another 48 hours. The psoralen-BSPL treated virus batch was considered non-infective if no CPE was observed after replating.

#### Testing psoralen-BSPL induced DNA damage by PCR

B virus gB primer set (5’-ACGATGCCCATGACGACCTTGC-3’ and 5’-GCGACGCGACCTTCTACGTCTG-3’) that amplifies a 1.9 bp fragment of B virus was used. The PCR reaction was performed by using the PCR HotStar Kit (Qiagen) and 3 μl of purified DNA in 20 μl volume. The amplification was performed on ABI Thermocycler 9600 using the following cycling conditions: 15 min 95˚C and 35 two step cycles of 20 sec at 95˚C and 40 sec at 65˚C. Then the PCR reaction products were run on 1% agarose gel along with the DNA marker to validate the presence of the PCR fragment of the expected size. The absence of the PCR fragment after amplification implied that DNA in the sample was damaged and could not be replicated.

#### Testing antigenicity of the psoralen-BSPL inactivated B virus by ELISA

B virus immunogens that were negative by the infectivity test and by PCR were tested by ELISA essentially as previously described [12]. Briefly, the immunogens were adsorbed to wells of a 96-well microplate at a final dilution of 1:100 in PBS, incubated for 1 hour at 37°C, washed 3 times with borate-buffered saline (BBS) containing 0.01% Tween 20, and blocked with Blotto (2.5% nonfat milk and 2.5% liquid gelatin in BBS). Six, 3-fold dilutions of a rhesus anti B virus positive standard serum starting from 1:50, were then incubated in the wells. After 3 cycles of washes, anti-human IgG alkaline phosphatase conjugate was added to the wells for 1 hour at 37°C. Wells were washed again and a dNPP substrate was added for 30 min at room temperature. Color intensity was then monitored in an ELISA-Reader at A_405_ nm.

### Production of mAbs

Production of mAbs to B virus was outsourced at the Monoclonal Antibody Facility, University of Georgia (MAF-UGA), of the Bioexpression and Fermentation Facility, University of Georgia, Athens, GA (Welfare Number A3437-01). The UGA protocol was reviewed by our Institutional Animal Care Committee (Assurance number A3914-01). Minimal animal suffering was assured. For collection of peritoneal macrophages, mice were anesthetized by isofluorane inhalation and euthanized by cervical dislocation.

Hybridomas were generated from mice inoculated with psoralen-BSPL-inactivated B virus or with a recombinant B virus gB preparation.

Mice inoculated intraperitonially with the B virus immunogen were first pretreated at time zero with Complete Freund's Adjuvant (CFA). Two days later they were injected with 500 μl of inactivated virus dilution containing approximately 10^6^ PFU of B virus (as determined before inactivation). Booster immunizations (same dose of immunogens without adjuvant) were then given at 3, 6, 9 and 12 weeks after first pretreatment immunization. Mice were bled for testing before immunization and 7 days after each booster injection until a satisfactory titer plateau was reached.

Mice were immunized intraperitonially with sterile solution of recombinant glycoprotein B (gB) prepared in PBS by inoculating 100 μg of recombinant gB emulsified in an equal volume of Complete Freund’s. This was followed 21 days later by a boost of 50 μg of gB mixed with an equal volume of Incomplete Freund’s adjuvant. The boost dosage was repeated every 21 days, with a blood sample taken 7 days after the boost, until a satisfactory titer plateau was reached.

### Testing reactivity of mAbs by ELISA

Antibody reactivities of the mAbs to B virus isolates from different macaque species and to a panel of human and nonhuman simplexviruses were tested by ELISA using infected cell lysates (inactivated) and recombinant proteins essentially as previously described [12, 13] except that the conjugate goat anti-human IgG–alkaline phosphatase was substituted with goat anti-mouse IgG–alkaline phosphatase.

### mAb competition ELISA (mAb-CE)

The mAb competition ELISA (mAb-CE) identifies antibodies to epitopes recognized by mAbs following natural infection. This test was performed essentially as previously described [22]. Briefly, antibody positive and antibody negative sera from macaques or humans and a “0” control in which buffer was added instead of sera, were first incubated (1 hr at 37°C) in B virus antigen coated 96-well microplates. The mAb of interest was incubated for another 1 hr at 37°C. After 3 cycles of washes with borate buffer + 0.05% Tween 20 the anti-mouse IgG-alkaline phosphatase conjugate was added and incubated (1 hr at 37°C). The wells were then washed again, incubated for 30 min at room temperature with the substrate. Color development was read in an ELISA reader as the optical density (OD) at A_405_ nm. Percent competition was calculated using the following formula: 100x[1-(OD test serum/OD negative serum)].

### The plaque reduction test for determination of neutralizing antibodies

Rhesus sera, positive and negative and a mouse negative serum were tested for neutralizing antibodies at 3-fold dilutions, 1:10 to 1: 2430. Some mAbs that were not concentrated or purified were tested at a 1:2 dilution and others that were concentrated or purified were tested at 3-fold dilutions (1:2 to 1:54). Serum dilutions were performed in the BSL2 lab. The 48-well Vero cell plate and the serum dilutions were transferred to the BSL-4 to for the addition of 25 μl containing 50 PFU (plaque forming units) B virus to 25 μl of the diluted serum.

The 50 μl virus + serum mixtures were incubated at 37°C incubator for 1 hour. A virus + PBS control was also introduced. After incubation 50 μL of virus + serum or virus + PBS mixtures were added to each well of the 48-well plate and incubated for 1 hr at 37°C in a humidified chamber containing 5% CO_2_ for virus adsorption. After additional 1 hr incubation, the virus+ serum inoculum was removed and 1%-methylcellulose was added to each well. The plate was incubated for 48 hours in a 37°C incubator to allow for plaque formation. After 48 hours, the methylcellulose from each well was removed and the wells were washed with PBS and fixed with 100% methanol. The plate was dunked out of BSL-4, washed, and then the cell cultures in the wells were stained with crystal violet (0.2%) to count the number of plaques. Percent neutralization of the virus was calculated relative to the virus + PBS control that measured the amount of plaques used in the test. The following formula was used: 100x[1-(S/B)], where S represents the number of plaques obtained from the serum + virus mixtures and B represents the number of plaques obtained with the virus + PBS control mixtures.

### Western blot analysis (WBA)

The WBA was performed according to standardized, CLIA-approved, diagnostic laboratory protocols as previously described [1, 13, 15].

### Immunoprecipitation

Immunoprecipitation was performed using the “Thermo Scientific Pierce Crosslink Immunoprecipitation Kit #26147 (Rockford, IL) per manufacturer’s protocol (http://www.thermoscientific.com/pierce).

### Mass spectrometry for protein identification (MS)

Protein bands of interest were excised from the gel. The excised gel pieces were washed first with dd-H_2_0 and subsequently with washing solution I (50% Methanol, 25mM Ammonium bicarbonate, pH 8.3), and washing solution II (50% Acetonitrile, 25mM Ammonium bicarbonate, pH 8.3). The washed gel pieces were finally dehydrated with 100% acetonitrile and dried under speed-vac. The dried gel pieces were either underwent trypsin digestion or kept at -80°C until they were treated with trypsin for the MS -peptide analysis. In brief, the gel pieces were incubated with appropriate amount of trypsin (Modified Trypsin Gold, Promega, Madison, WI) in proteaseMAX surfectant (Promega, Madison, WI) at 37°C for 2~ 3 hours. After incubation, the digested peptides were extracted with 2.5% of trifluoroacetic acid. The extracted peptides were further purified and concentrated by ZipTip, a C18 micro-reverse phase resin tip (Millipore, Billerica, MA) according to the manufacturer’s protocol.

Extracted peptides were then analyzed by 4800 MALDI Tof/Tof tandem mass spectrometer (AB Sciex, Framingham, MA) with MS/MS tandem mode. Protein identifications were performed using Mascot search engine (Matrix Science Inc, Boston, MA) against Swiss Pro or NCBI protein database.

### Statistical analysis

One-way ANOVA with Bonferroni's Multiple Comparison Test was performed to evaluate differences in mAb reactivity using GraphPad Prism™ version 5.00 software, San Diego California USA, http://www.graphpad.com.

## Results

### Characterization of mAbs

Table 1 lists descriptions and classification of each of the 10 mAbs used. From these, one mAb (#6), was induced using recombinant B virus gB, while the others were generated using inactivated rhesus B virus (strain E2490).

**Table 1:** Overall summary of mAbs discussed in this publication and some of their characteristics.

mAb Serial Number	mAb	Ig Class or Subclass	Category	tELISA	Rec-ELISA	WBA	IP/MS	Neutraliziing
1	12F5.C1	IgG1	I	**POS**	NEG	**POS**	VP13/14	NEG
2	12G9.G5	IgG2a	I	**POS**	NEG	NEG	gE and gI	NEG
3	5E10.C10	IgG1	II	**POS**	BV-gB	NEG	ND	NEG
4	7F7.G7	IgG2b	II	**POS**	BV-gB	NEG	ND	NEG
5	18D10.F2.A4	IgA	III	**POS**	BV-gB HSV1-gB	NEG	ND	NEG
6	7H1.G5	IgG1	III	**POS**	BV-gB HSV1-gB	NEG	gB	NEG
7	5D10.C9	IgG1	III	**POS**	BV-gB HSV1-gB	NEG	ND	NEG
8	7G9.E3	IgG1	III	**POS**	BV-gB HSV1-gB	NEG	ND	NEG
9	2G12.D12.D4	IgG2a	IV	**Weak**	BV-gD	**POS**	ND	NEG
10	6E10.D7	IgG2b	IV	**Weak**	BV-gD	**POS**	ND	NEG

Ig, immunoglobulin; POS, positive; NEG, negative; BV, B virus; HSV1, herpes simplex virus 1; gB, glycoprotein B; gD, glycoprotein D; WBA, western blot analysis; IP/MS, immunoprecipitation and mass spectrometry analysis; ND, not done.

**Fig 1:**
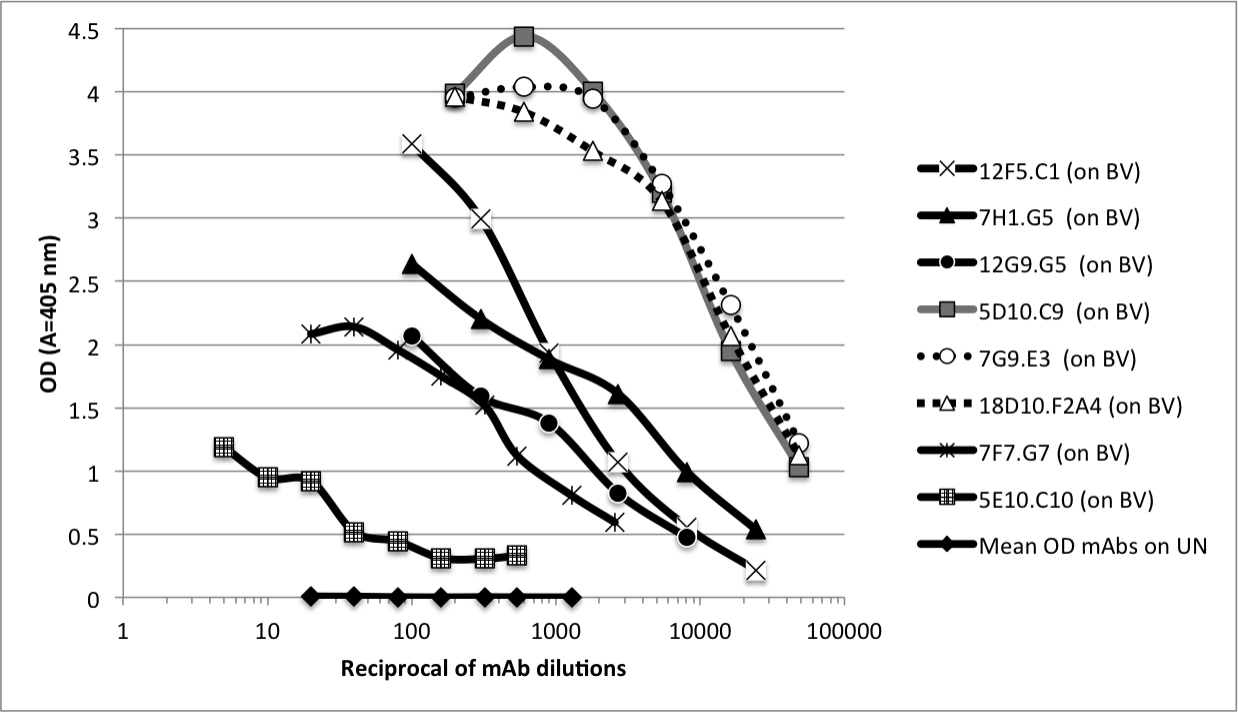
Titration of mAbs by ELISA on wells coated with B virus infected cell lysates (BV) and uninfected cell lysates (UN). The OD values represent average values from duplicate tests. Control results were obtained by titrating all eight mAbs at the indicated dilutions on the UN antigen. OD values represent mean values of all mAbs at each dilution point.

ELISA results of mAbs 1–8 (Summarized in Table 1) are also shown as titration curves using B virus coated wells and on control uninfected Vero cell lysates (UN) (Fig 1). Results of mAbs 9 and 10 are not shown because of their weak response even at low dilution.

The majority of mAbs (#3–8) reacted with recombinant gB proteins. The mAbs #9 and #10 reacted with B virus gD (Tables 1 and 2), while mAbs #1and #2 showed no detectable reactivity by Rec-ELISA with B virus recombinant glycoproteins gB, gC, gD, gE, gG and gI.

**Table 2:** Reactivity of mAbs with B virus (BV) or HSV-1 glycoproteins.

			Recombinant Proteins			
mAb Serial Number	Category	mAb Name	BV-gB	BV-gD	HSV1-gB	HSV1-gD
1	**I**	12F5.C1	1.5	1.1	0.5	0.7
2		12G9.G5	1.1	1.5	0.6	0.6
3	**II**	5E10.C10	**14.4**	0.6	0.8	0.9
4		7F7.G7	**163.7**	1	**10.8**	0.4
5	**III**	7H1.G5	**435.6**	1.8	**165.3**	0.9
6		5D10.C9	**140.4**	0.8	**127.8**	0.6
7		7G9.E3	**596.3**	1	**469.6**	3.1
8		18D10.F2.A4	**164.7**	0.8	**148**	1.6
9	**IV**	2G12.D12.D4	1.6	**245.8**	4.2	1.1
10		6E10.D7	0.4	**108.5**	1.8	1.6

Numbers in table represent P/N ratio values (OD values obtained with the virus glycoprotein divided by the OD value obtained with a nectin control). Bold numbers represent positive results. In this experiment P/N values higher than 4.5 were considered as positive.

**Fig 2:**
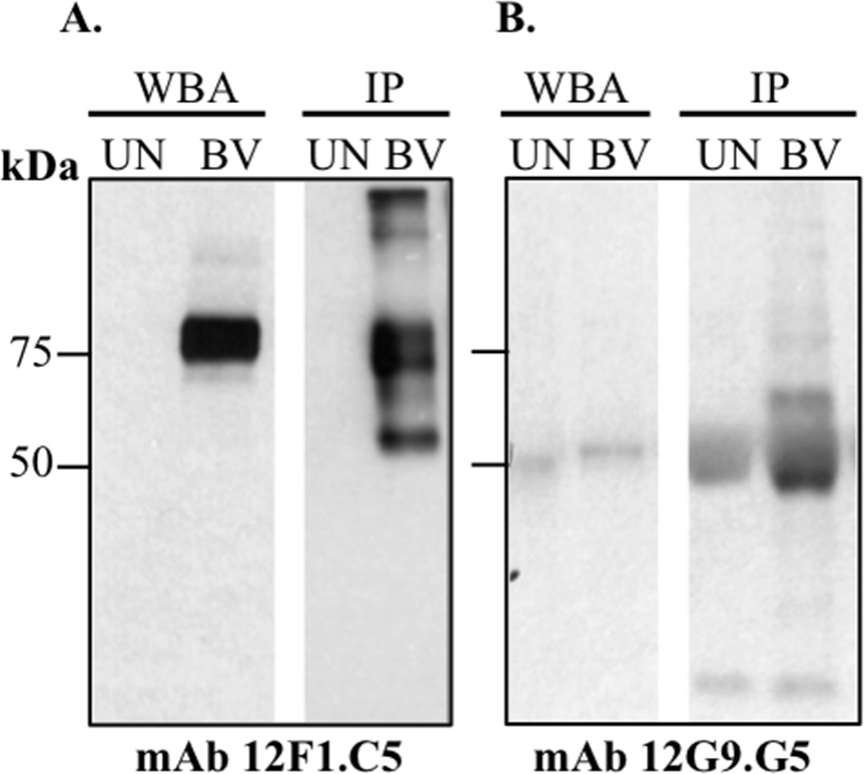
Analyses of specificity of Category-I mAbs (12F1.C5 and 12G9.G5) by western blot analysis (WBA) and immunoprecipitation (IP) using SDS PAGE-fractionated B virus infected cell lysates. (**A**) mAb 12F1.C5 demonstrated recognition of a 75 kDa protein by WBA and IP: (**B**) the mAb 12G9.G5 was negative by WBA but demonstrates recognition of ~45 kDa to 60 kDa proteins by IP. UN, Vero cells lysates; BV, B virus infected cell lysates.

By WBA, mAb #1 reacted with a 75 kDa protein (Fig 2A) and the anti-gD #9 and #10 reacted as expected with a 50 kDa protein (not shown). All other mAbs were negative by WBA. By immunoprecipitation, mAb #1 precipitated a 75 kDa protein (correlating with WBA result) (Fig 2A), which was identified by MS as the B virus tegument protein VP13/14. The mAb #2 (negative by WBA) immunoprecipitated bands with molecular weights spanning from ~45 kDa to ~60 kDa (Fig 2B). These bands were identified by MS as gE and gI. The specificities of mAbs #1–8 were identified by ELISA using five B virus isolates and six (two human and four non-human) primate simplexviruses (Table 3). None of the 10 mAbs neutralized B virus.

**Table 3:** The relative reactivity of mAbs to viruses of the simplexvirus group was tested by tELISA.

mAb Serial No	Cat	mAb	BVL	BVR	BVC	BVP	BV10	HP2	SA8	HVL	HVM	H1	H2
1	**I**	12F5.C1	**100**	**91**	1	5	**100**	0	1	1	0	1	6
2		12G9.G5	**100**	**106**	6	3	**169**	2	2	6	3	5	9
3	**II**	5E10.C10	**100**	**57**	**113**	**86**	**95**	**85**	29	**66**	**37**	4	9
4		7F7.G7	**100**	**59**	**61**	1	**103**	**66**	9	**40**	12	9	6
5	**III**	5D10.C6	**100**	**98**	**103**	19	**100**	**76**	14	**82**	**52**	11	23
6		7G9E3	**100**	**95**	**102**	9	**79**	**119**	28	**117**	13	15	14
7		7H1.G5	**100**	**114**	**112**	**128**	**114**	13	4	**115**	**53**	16	**41**
8		18D10.F2.A4	**100**	**90**	**93**	**97**	**100**	**81**	60	87	**76**	**84**	**58**

Results were obtained from triplicate experiments. Numbers represent the mean percent reactivity relative to the rhesus B virus strain lab strain that was used for immunizing the mice. Bold numbers ≥50% represent strong reactivity. Numbers >30% to 50% indicate intermediate reactivity. Numbers <30% indicate low or no activity. Cat, Category; BVL, BV lab strain; BVR, BV strain RRN6; BVC, BV strain CY8166; BVP, BV strain PRN; BV10, BV strain 10R; HP2, HVP2; HVL, Langur herpesvirus; HVM, Mangabey herpesvirus; H1, HSV-1; and H2, HSV-2.

### Categorization of the mAbs

Based on the reactivity and specificity of the mAbs each was classified into one of four categories:

**Category-I** includes mAbs #1 and #2 that recognized rhesus B virus isolates and Japanese B virus isolates, but not B virus isolates from pigtail or cynomolgus macaques, or any other tested simplexvirus.

**Category-II** includes mAbs #3 and #4 reactive with B virus gB, but non-reactive or poorly reactive with HSV-1 gB. Monoclonal antibody #3 reacted to each B virus isolate, however, mAb #4 did not react with isolates from pigtail macaques, although it did react with the others. These two mAbs reacted with some of the simian simplexviruses, but did not react with the human simplexviruses (HSV-1 and HSV-2) (Tables 1, 2 and 3). This classification indicates that although mAb #3 and #4 were similar, they recognized different epitopes of the same protein.

**Category-III** includes mAbs #5–8 that reacted with B virus isolates as well as with other simplexviruses including HSV-1 and HSV-2. These mAbs also reacted with recombinant B virus gB and HSV1-gB (Tables 2 and 3).

**Category-IV** includes mAbs #9-#10 that reacted poorly in ELISA with solubilized B virus antigen, reacted strongly with B virus gD but not with HSV-1-gD (Table 2).

### Pooled macaque and human sera analyzed by the mAb-CE

Serum pools from rhesus and cynomolgus macaques, as well as from humans confirmed as B virus antibody-negative or -positive, along with serum pools from HSV-1 and HSV-2 antibody-positive humans were tested by the mAb-CE against mAbs #1–8. As shown in Table 4, binding of Category-I mAbs to B virus antigen was blocked (competed) only by the rhesus and human B virus antibody-positive serum pools and not by B virus antibodies from cynomolgus or human HSV-1&-2 antibodies. These results correlated with the whole-virus ELISA results shown in Table 3.

**Table 4:** mAbs blocked by pooled macaque and human sera as determined by the mAb-CE.

			Sera used for competition in the mAb-CE							
Cat	mAb Serial No	mAb	Rh Neg	Rh anti BV	Cyno Neg	Cyno anti BV	Hu Neg	Hu anti BV	Hu anti HSV-1	Hu anti HSV-2
**I**	**1**	**12F1.C5**	8.0	**57.6**	-1.7	4.4	13.5	**83.7**	-19.6	-30.5
	**2**	**12G9.G5**	1.8	**55.0**	-13.1	-55.5	17.3	**58.0**	0.5	-14.9
**II**	**3**	**5E10.C10**	27.0	**57.9**	19.0	**66.0**	34.8	**79.9**	**84.1**	**86.9**
	**4**	**7F7.G7**	18.2	**77.8**	1.1	**81.3**	40.4	**92.4**	**92.0**	**92.4**
**III**	**5**	**18D10.F2.A4**	3.2	**93.0**	-14.8	**77.1**	16.6	**75.2**	**72.7**	**62.3**
	**6**	**7H1.G5**	-39.9	**74.7**	0.6	**72.5**	-19.7	**83.3**	**91.8**	**86.9**
	**7**	**5D10.C6**	-5.2	**93.0**	9.5	**94.9**	4.9	**77.1**	**93.4**	**83.9**
	**8**	**7G9.E3**	0.8	**94.7**	3.0	**92.2**	27.7	**82.2**	**94.2**	**88.2**

Numbers represent percent competition values. Bold numbers highlight positive competition results. For this experiment competition values ≥50% were considered positive.

Rh Neg, B virus antibody negative rhesus serum; Rh anti BV serum, B virus antibody positive rhesus serum; Cyno Neg, B virus antibody negative cynomolgus serum; cyno anti BV, B virus antibody positive cynomolgus serum; Hu Neg, herpes virus antibody negative human serum; Hu anti BV, B virus antibody positive human serum; Hu anti HSV-1, HSV-1 antibody positive human serum, Hu anti HSV-2, HSV-2 antibody positive human serum.

Binding of Category-II mAbs to B virus antigen was blocked by B virus antibody-positive human and macaque serum pools, as expected, but these were also blocked by HSV-1&-2 antibody-positive serum pools, despite lack of reactivity with HSV antigens in the whole-virus ELISA (Tables 2 and 3).

Binding of Category-III mAbs to B virus antigen was blocked by B virus antibody-positive human and macaque serum pools and also by HSV-1&-2 antibody-positive serum pools, which correlated with results shown in Tables 2 and 3, indicating that these mAbs reacted with simplexvirus group-specific epitopes.

### Individual rhesus and cynomolgus macaque serum samples analyzed by the mAb-CE

B virus antibody-positive rhesus and cynomolgus macaque sera (tELISA titer >5000) were tested by the mAb-CE against two “Category-I mAbs (12F5.C1 and 12G9.G5), one Category-II mAb (7F7.G7), and against three Category-III mAbs (7H1.G5, 5D10.C6, and 18D10.F2.A4).

**Fig 3:**
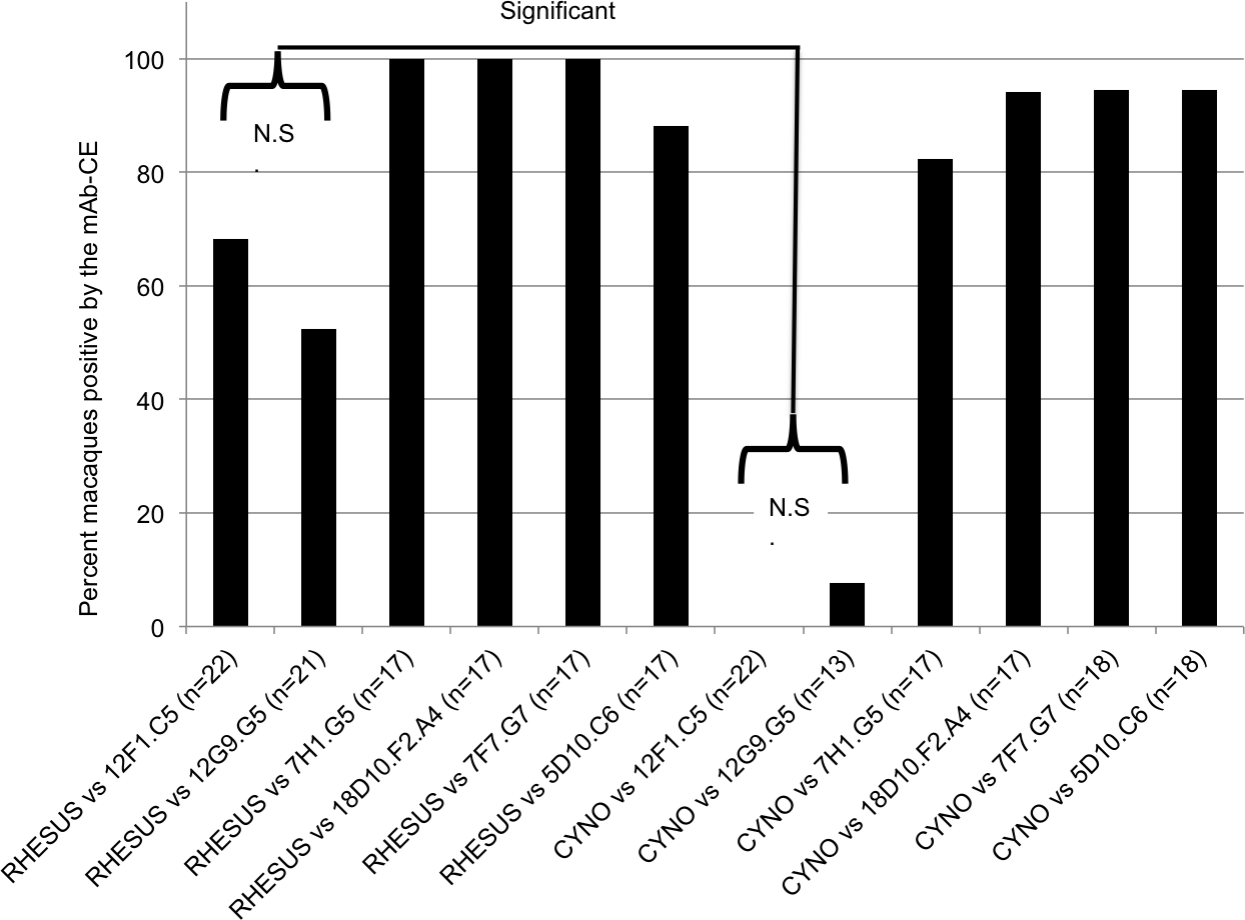
Percent competition of rhesus and cynomolgus B virus antibody-positive sera by the mAb-CE. Individual rhesus and cynomolgus macaque serums competed against two Category-I mAbs (12F5.C1 and 12G9.G5) for reactivity with B virus infected cell lysates in mAb-CE, compared to reactivity of one Category-II mAb (7F7.G7), and three Category-III mAbs (7H1.G5, 5D10.C6, and 18D10.F2.A4). The numbers of animals (n) tested in each group are indicated. Values ≥50% competition were regarded as positive competition. Significant, statistically significant (p≤0.05). N.S., statistically not significant (p>0.05).

The percent of individual B virus positive rhesus and cynomolgus macaques that were positive by the mAb-CE was calculated and presented in Fig 3.

Approximately 80–100% of the rhesus and cynomolgus macaque sera tested competed against the Category-II and Category-III mAbs. As expected (Table 4), the Category-I mAbs significantly competed by rhesus macaque sera but not by cynomolgus sera (P<0.05). Only 68% of the rhesus sera, however, competed against mAb #1 and 52% of the rhesus sera competed against mAb #2, indicating that not all rhesus macaques may have developed antibodies against the specific epitopes recognized by mAb #1 and mAb #2. However, this lower competition reactivity was not statistically significant when compared to the higher competition reactivity that was found for Category-II and Category-III mAbs. In a separate experiment we tested the abilities of individual B virus antibody positive macaque sera from different tELISA titer groups (<50 and <500, <5000 and ≥5000 (EU)) to compete with four mAbs (two Category-I mAbs, 12F5.C1 and 12G9.G5, and two Category-III mAbs, 7H1.G5 and 18D10.F2.A4). Results summarized in Table 5 confirm the lack of competition of cynomolgus sera with the Category-I mAbs and also show that those macaques with antibody titers lower then 500 EU failed to compete significantly with any of the tested mAbs.

**Table 5:** Percent of rhesus and cynomolgus macaques positive by the mAb-CE.

mAbs	12F5.C1		12G9.G5		7H1.G5		18D10.F2.A4	
Competed with	Rhesus sera	Cyno sera	Rhesus sera	Cyno sera	Rhesus sera	Cyno sera	Rhesus sera	Cyno sera
tELISA group ≤50 EU	0*	0^<^	8.3*	0^<^	0*	0^<^	0*	6.7^<^
tELISA group 50 to <500 EU	0*	5.9^+^	0*	7.6^+^	7.7*	35.3^+^	0*	5.9^+^
tELISA group 500 to <5000 EU	62.6^	25^•^	20^#^	0^#^	81.5^	58.3^•^	100^	91.7^•^
tELISA group 5000 to >5000 EU	94.1^+^	11.8^+^	80^#^	0^#^	100^+^	76.5^+^	100^+^	88.2^+^

Individual rhesus and cynomolgus macaque sera from different tELISA titers groups (≤50 to ≥5000 EU) were competed against the two “Category I” mAbs (12F5.C1 and 12G9.G5), and for comparison against two “Category III” mAbs (7H1.G5 and 18D10.F2.A4) in the mAb-CE. Numbers indicate percent of macaques positive by mAb-CE. A level of ≥30% competition was regarded as positive competition. The number of serum sample (n) in each of the experimental groups is indicated by the following symbols:

^#^, n = 5

^•^, n = 12

*, n = 14

^<^, n = 15

^, n = 16

^+^, n = 17.

### Human serum samples analyzed by the mAb-CE

Twenty-two individual human serum samples and three pooled control serum samples (human negative, human HSV-1 antibody-positive, human HSV-2 antibody-positive) were tested by the mAb-CE against two mAbs of Category-I and mAb #6 of Category-III (Table 6). Twenty-one of these serum samples were received at the National B virus Resource Center for B virus-antibody evaluation.

**Table 6:** mAbs competed by human sera as determined by the mAb-CE.

	mAb-CE (% competition ± SD)			B Virus or HSV Status			
HUMAN SERA	12F5.C1 mAb	12G9.G5 mAb	7H1.G5 mAb	B virus	HSV-1	HSV-2	ELISA Titer (EU)
Hu Neg (Pool)	2.2±16	15.2±30	-13.2±9.2	N	N	N	N
Hu Neg (VM)	8.6±8.6	13.9±8.9	12.8±3.6	N	N	N	N
Hu BV-Pos + VM	**90.9±1.8**	**61.9±0.4**	**95.6±0.2**	NT	NT	NT	NT
Hu BV-Pos + anti-HSV1	**91.0±0.1**	**66.4±4.4**	**92.0±0.1**	NT	NT	NT	NT
Hu BV-Pos + anti-HSV2	**92.3±0.3**	**59.4±1.3**	**90.8±0.6**	NT	NT	NT	NT
anti-HSV1 (Pool)	-19.6	0.5	**91.8**	N	P	N	10,000
anti-HSV2 (Pool)	-30.5	-14.9	**86.9**	N	N	P	3,500
hI (aS)	15.1±0.2	9.0±8.4	17.9±0.5	P	P	N	2,000
hB (aS)	25.5±4.4	13.0	**30.9±8.3**	P	N	N	10,000
hN (aS)	-86.9	NT	**77.2**	P	P	N	NT
hL (aS)	16.14	2.86	36.67	Ind	N	N	25,000
hO (aS)	2.4	-0.3	**87.7**	P	P	P	10,000
hP (aS)	5.6	-3.7	**96.7**	N	P	P	10,000
hR (aS)	19.0±5.8	-5.0±32.0	**98.0±0.4**	P	P	N	30,000
hS (aS)	27.0±12.8	8.0±30.6	**51.0±0.8**	P	N	P	900
hK (aS)	20.3	33.0	**54.5**	Ind	P	N	60
hT (aS)	1.5	21.3	**100.0**	P	N	N	80,000
hM (aS)	8.27	11.88	**73.05**	Ind	Ind	Ind	100
hJ (aS)	-102.3	NT	**30.9**	P	Ind	Ind	50
hA (Sy)	**89.8±3.7**	**79.2±3.7**	**89.5±8.1**	P	N	N	3,000
hG (Sy)	**55.8±0.8**	**52.5±8.2**	**95.4±3.1**	P	N	N	2,000
hC (Sy)	-41.4	14.0	-37.9	P	N	N	250
hF (Sy †)	-67.5	-52.9	**43.1**	P	N	N	250
hH (Sy †)	-47.7	NT	-28.7	P	N	N	600
hD (Sy †)	-47.7	NT	**60.3**	P	P	N	NT
hE (Sy †)	-53.1	-52.1	**97.2**	P	P	N	30,000
hU	4.2	NT	-3.3	N	N	N	N
hW	-68.5	NT	-19.1	N	N	N	N

Human sera were competed against the two Category-I mAbs (12F5.C1 and 12G9.G5) and against one Category-III mAb (7H1.G5). Numbers indicate percent competition values. For the sake of the discussion, competition values of 30% or higher were considered as positive (bold numbers). Standard-deviation values (±SD) were calculated from replicate measures. Controls consisted of a pool of herpes virus-negative human sera (Hu Neg (Pool)), a serum sample from a herpes virus-negative individual (Hu Neg (VM)), an anti- HSV-1 positive serum pool (anti-HSV-1 (Pool)), an anti-HSV-2 positive serum pool (anti-HSV-2) (Pool)) and a human B virus positive serum (BV-Pos) spiked with the anti-HSV1 and anti-HSV2 pools diluted to contain 1000 EU of antibodies quantified using ELISA. Some of the control results are identical to the results in Table 4 and were placed here for the sake of comparison.

N, negative; P, positive; Ind, indeterminate; NT, not tested;, aS, asymptomatic; Sy, symptomatic

†, deceased.

Of the 21 individuals, 12 were monitored post macaque exposure and seroconverted (asymptomatic) while seven were from patients with clinically apparent disease (symptomatic), and two had no laboratory or clinical signs of infection (negative). Of the seven with acute disease four succumbed as a result of B virus zoonotic infection (Table 6).

The confirmatory laboratory results of “B virus or HSV antibodies” are shown in the last four columns of Table 6. The antibody titers (specific and/or cross-reactive) are shown in the last column. Anti-HSV-1 and anti-HSV-2 pooled sera competed as expected only with the 7H1.G5 mAb that recognized group-common epitopes. Negative sera did not compete non-specifically with any of the mAbs.

Eleven of 12 B virus antibody positive, post-exposure sera competed only with the 7H1.G5 mAb. Of the 11 human sera, three (hB, hL, and hT) had no detectable HSV antibodies, thus, competition in the absence of HSV antibodies suggests B virus infection. The mAb-CE competition results obtained with the other eight B virus positive sera that were also positive for either HSV-1 or HSV-2 antibodies could not be interpreted as a confirmation for a B virus infection. Three of the seven symptomatic patients recovered from disease. No serum samples from the four deceased patients competed with Category-I mAbs, but three competed with the 7H1.G5. One of the three sera was HSV antibody-negative and therefore the competition likely resulted from the presence of B virus antibodies. Of the three sera from symptomatic patients who recovered form zoonotic B virus disease, one failed to block all three mAbs. Serum samples from the two other patients competed with all three mAbs. The competition observed with the two B virus specific Category-I mAbs was interpreted as further evidence of B virus specific antibodies in these two serum samples. In addition, the competition of these two serum samples with mAb #6, indicates that the B virus infection in these individuals also induced antibodies to group-common epitopes.

To rule out the possibility that a negative competition against the Category-I mAbs is due to coexisting HSV-1 or HSV-2 antibodies, antibody-negative sera were spiked with HSV-1 and/or HSV-2 antibody positive sera. Analysis of mAb-CE data indicate that HSV antibodies did not interfere with the B virus antibody-specific competition with the Category-I mAbs (Table 6).

## Discussion

The current diagnostic tests are sufficient for B virus diagnosis in macaques, however, in humans; B virus diagnosis is confounded because of the cross-reacting human simplexviruses (HSV-1 & -2). Therefore, there is a need to develop improved assays for differential B virus diagnosis, which requires B virus specific reagents. To achieve this goal we produced mAbs to B virus antigen. Our present study resulted in the production of a novel panel of mAbs, which was used to identify specific immunoreactive epitopes of B virus proteins for the first time since the discovery of B virus over 80 years ago. The mAbs identified epitopes of five B virus proteins: gD, gI, gE gB and VP13/14. Neutralizing experiments revealed that none of the panel of mAbs neutralized B virus. Envelope virus gD binds to host cell receptors and triggers B virus and HSV entry into target cells [26–30]. The inability of B virus gD-specific mAb to neutralize B virus infection of Vero cells may support our previously reported observation that B virus has a unique gD-independent entry pathway. It is likely that this particular mAb-detected epitope is not essential for virus entry. The B virus neutralizing ability of the anti-gD mAb, will be examined in B virus entry-resistant cells bearing a single gD receptor, human nectin-1 or -2 [28, 29].

The mAbs 12F5.C1, 2G12.D12.D4, 6E10.D7 appeared to recognize linear epitopes, while the other mAbs likely recognized conformational epitopes. In comparison with others, we obtained a greater proportion of mAbs reactive with conformational epitopes possibly because we used immunogens in which protein conformation was partially or fully preserved [21]. B virus VP13/14 was identified as the target protein for the B virus specific mAb 12F5.C1. Of note, this inner gamma 2 tegument protein, a structural protein, induced antibodies in mice after inoculation of an inactivated virus preparation as well as in macaques and humans after natural infection. Although it is widely accepted that antibodies to surface proteins are prevalent markers of viral infections, induction of antibodies to inner structural and nonstructural proteins and their importance for early diagnosis of infection was reported for other viruses such as retroviruses, flaviviruses and enteroviruses [31–34].

B virus gE and gI were identified as target proteins for mAb 12G9.G5. Because this mAb did not react with recombinant gE or gI and because glycoproteins gE and gI are known to form complexes, this mAb is likely directed to an epitope located at the junction of the gE/gI complex [35, 36]. Both 12F5.C1 (anti-VP13/14) and 12G9.G5 (anti-gE/gI), Category-I mAbs, are strain specific because they reacted with B virus isolates from both rhesus and Japanese macaques, but did not react with B virus isolates from pigtail and cynomolgus macaques. A similar finding for a mAb specific for B virus isolates was previously reported [16]. These results support previous findings indicating that rhesus B virus and Japanese macaque B virus isolates are more distantly related to cynomolgus B virus and pigtail B virus isolates [37]. These mAbs can be used to establish the species of specific macaques involved in zoonotic infections in absence of information regarding a known exposure, as often is the case.

The B virus stain specificity of the Category-I mAbs was also confirmed by the mAb-CE test in which these mAbs were competed by rhesus macaque B virus antibodies and not by cynomolgus macaque B virus antibodies or human anti HSV antibodies. Category-II mAbs reacted with each B virus isolate as well as with certain non-human primate simplexviruses, but did not react with the human simplexviruses. Interestingly, however, when tested by the mAb-CE test, these mAbs were competed by HSV-1/HSV-2 antibodies. Blocking binding of the Category-II mAbs to their B virus-specific epitopes may be caused by steric hindrance due to binding of the HSV-common antibodies to a nearby cross-reacting epitopes [5, 21]. However, epitope mapping of the Category-II mAbs may provide the identification and synthesis of B virus-specific epitopes that can be used in peptide-based assays for differential serological diagnosis of B virus infection in humans with pre-existing HSV-1/HSV-2 antibodies.

Category-III mAbs recognize simplexvirus group-common epitopes, facilitating studies to better understand simplex virus conserved epitopes that are found in human and nonhuman primate herpesviruses (Tables 2 and 3). These antibodies are invaluable for enhancing our understanding the identity and function of conserved epitopes in human and nonhuman primate simplex herpesviruses. As expected, Category-III mAbs that recognize group-common epitopes were competed in the mAb-CE by B virus antibody-positive sera as well as by the anti-HSV-1 and anti-HSV-2 sera.

Here we show that the mAb-CE can be used to confirm the presence of B virus antibodies in macaques by employing Category-II and Category-III mAbs since there is no evidence, thus far, of the presence of antibodies to other simplexviruses in macaques that may confound the diagnosis by steric hindrance [1, 5, 12, 22]. Using Category-II and Category-III mAbs in the mAb-CE test with serum samples from humans with mixed B virus, HSV-1 and/or HSV-2 antibodies is likely to be challenging to interpret due to cross-reactivity or steric hindrance caused by antibodies to group-specific epitopes (Table 6, patients hE, hD, hM, hK, hS, hR, hP, hO, hN). Positive competition results against these mAbs with serum from B virus infected individuals in the absence of antibodies to HSV-1 and HSV-2 may be an indication of a true B virus infection (Table 6; patients hB, hL, hT and hF). However, positive competition results against the Category-I mAbs as obtained with two human sera (hA and hG), represent a true B virus infection. We have shown in a spiked control experiment (Table 6) that this result will not be affected by the presence of antibodies to HSV-1 or HSV-2. Because these mAbs are reactive only with certain B virus isolates, some B virus infections with other isolates will likely be missed. Alas, for the humans that die shortly after infection, antibodies may have not developed or were below the limit of detection. It is also generally appreciated that antiviral drug treatment may also weaken the antibody response in some individuals. Further, some epitopes may not be immunogenic in certain individuals because of the genetic diversity of the HLA system in the human population [21, 38]. These challenges remain to be addressed. Category-IV anti-gD mAbs were specific for B virus and reacted with linear epitopes that when identified will be employed in specific peptide-based assays.

In conclusion, a novel panel of mAbs was described that demonstrated B virus specific reactivity differentiable fromll simplexvirus group reactivity.

A rare collection of patient sera enabled us to perform this study to better understand antibody responses to zoonotic B virus infections and to potentially enhance identification of these deadly infections.

We have shown that the Category-I mAb can be used in competition ELISAs to identify zoonotic B virus infections in humans even in the presence of HSV antibodies.

The selective strain specificity of these mAbs limit their general use for diagnosis of B virus infections in humans currently, but may be of value for strain identification of field isolates and for studying strain-specific pathogenesis.

These findings further our aim of developing assays that enable unequivocal differential diagnosis of zoonotic B virus infection. To fully achieve our aim more mAbs have to be produced to complete the range of desired specificities. In addition, epitope mapping of these mAbs and of those that will be produced in the future will have to be carried out. B virus specific epitopes, thus identified, will be used for the development of accurate peptide-based assays for differential serological diagnosis of B virus infections in humans.
